# Anastomosing haemangioma with fatty changes in the perirenal space: a lesion mimicking liposarcoma

**DOI:** 10.1259/bjrcr.20170022

**Published:** 2017-11-01

**Authors:** Naotaka Kishida, Kazuhiro Sentani, Hiroaki Terada, Yukiko Honda, Keisuke Goto, Yui Hatanaka, Kenichi Kohashi, Yoshinao Oda, Jun Iwata, Wataru Yasui, Shunsuke Shinmei, Tetsutaro Hayashi, Jun Teishima, Akio Matsubara, Yuko Nakamura, Makoto Iida, Kazuo Awai

**Affiliations:** ^1^Department of Diagnostic Radiology, Institute of Biomedical and Health Sciences,Hiroshima University, Minami-ku, Hiroshima, Japan; ^2^Department of Molecular Pathology, Institute of Biomedical and Health Sciences,Hiroshima University, Minami-ku, Hiroshima, Japan; ^3^Department of Urology, Institute of Biomedical and Health Sciences, HiroshimaUniversity, Minami-ku, Hiroshima, Japan; ^4^Department of Anatomic Pathology, Pathological Sciences, Graduate School of Medical Sciences, Kyushu University, Higashi-Ku, Fukuoka, Japan; ^5^Department of Diagnostic Pathology, Kochi Health Sciences Center, Kochi, Japan

## Abstract

Anastomosing haemangioma is a rare subtype of capillary haemangioma. Pathologically, anastomosing haemangioma presents with anastomosing sinusoidal capillary-sized vessels in an architecture reminiscent of the splenic parenchyma. Its anastomosing architecture pathologically can lead to concern for angiosarcoma. Many cases of anastomosing haemangioma, which often occurred in the retroperitoneum, were well circumscribed, hyperdense on plain CT, revealed avid contrast enhancement and some of them exhibited fatty changes. In cases of tumours with fat of retroperitoneal occurrence, images frequently do not allow for easy differentiation from liposarcoma. Although anastomosing haemangioma with fatty changes and liposarcoma can be difficult to differentiate, no previous report has addressed this diagnostic difficulty. We have encountered a case of anastomosing haemangioma with fatty changes occurring in the perirenal space that was difficult to differentiate from liposarcoma. With retroperitoneal tumours accompanied by fatty changes and including a strongly enhanced area, the possibility of anastomosing haemangioma—which is a benign tumour—may also be considered. In such cases, biopsy is an effective means of diagnosis.

## Case presentation

The patient was a 75-year-old woman without a chief complaint. Abdominal ultrasound for cancer screening revealed a retroperitoneal hypoechoic mass measuring 26 × 22 × 18 mm in the perirenal space, at the level of the left renal upper pole. Subsequently, she was referred to our hospital for examination.

## Investigations, Imaging findings

**Figure 1.**
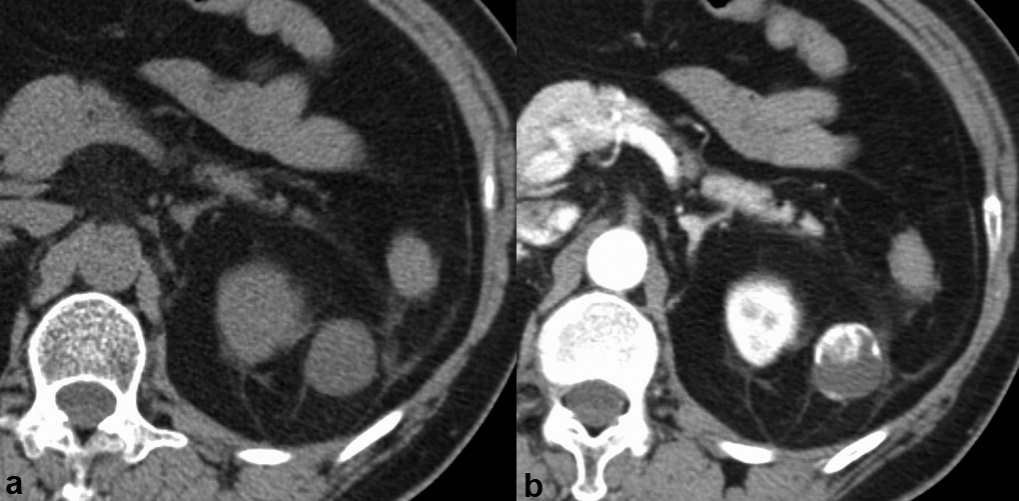
CT images of retroperitoneal anastomosing haemangioma. (a, b) The ventral side of the mass exhibited avid contrast enhancement equivalent to the renal cortex in the corticomedullary phase.

**Figure 2.**
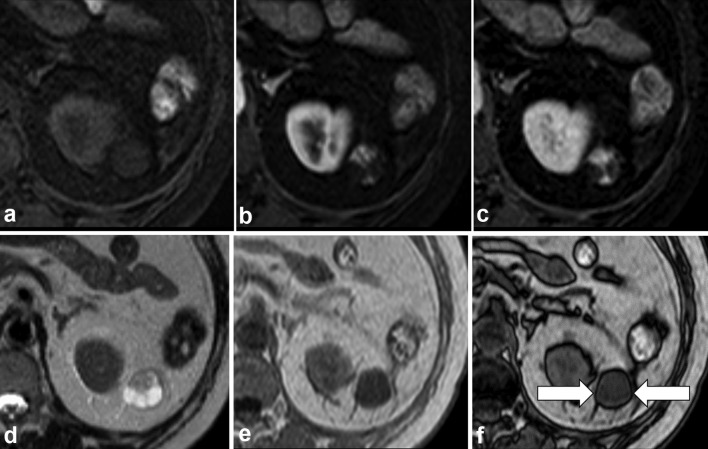
MR iImages of retroperitoneal anastomosing haemangioma. (a, b) The ventral side of the mass exhibited avid contrast enhancement equivalent to the renal cortex in the corticomedullary phase. (c) The ventral side of the mass exhibited prolonged enhancement. (d) The ventral side of the mass was heterogeneous and lower in intensity than the cerebrospinal fluid in the *T*_2_ WI. (e, f) On the dorsal side of the mass, there appeared to be a decrease in intensity from in-phase to out-of-phase in *T*_1_WI (arrows); however, it was impossible to conclude preoperatively whether fat was present in the mass.

Abdominal CT and MRI were performed. The mass was well circumscribed, with fat stranding in the surrounding adipose tissue, and demonstrated heterogeneous enhancement (Figure 1a,b). The ventral side of the mass exhibited avid contrast enhancement equivalent to the renal cortex in the corticomedullary phase, with prolonged enhancement (Figure 2a–c). The ventral side of the mass was heterogeneous and lower in intensity than the cerebrospinal fluid in *T*_2 _weighted image (WI) (Figure 2d) and of low intensity in *T*_1_WI (Figure 2e). The dorsal side of the mass exhibited low intensity in *T*_1_WI and high intensity in *T*_2_WI and, although almost no enhancement area was observed, overall, it was believed to be a poorly enhanced cystic structure. Plain CT did not indicate a fat component within the mass. On the dorsal side of the mass, there appeared to be a decrease in intensity from in-phase to out-of-phase in *T*_1_WI; however, it was impossible to conclude preoperatively whether fat was present in the mass (Figure 2e, f).

**Figure 3.**
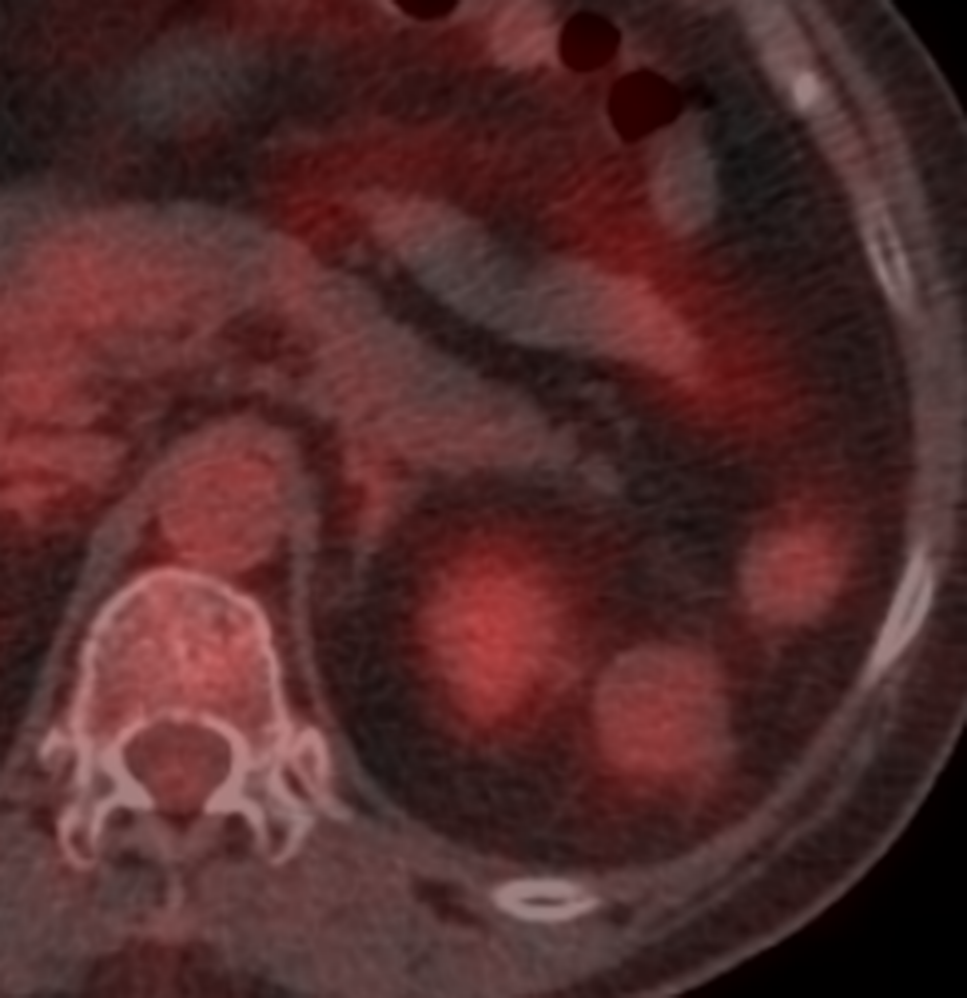
^18^F-fludeoxyglucose positron emission tomography/CT image of retroperitoneal anastomosing haemangioma. Minor accumulation was observed in the mass [standardized uptake value maximum (SUV_max_) 2.5].

On ^18^F-fludeoxyglucose positron emission tomography/CT (FDG-PET/CT), minor accumulation was observed in the mass [standardized uptake value maximum (SUV_max_) 2.5] (Figure 3). No significant accumulation was observed in other regions.

## Differential diagnosis

Owing to avid contrast enhancement, paraganglioma was considered in the differential diagnosis; however, on ^123^I-metaiodobenzylguanidine scintigraphy, significant accumulation was not observed. Therefore, we thought that the possibility of paraganglioma was low.

Dedifferentiated liposarcoma was the probable diagnosis because of the following reasons:

- Liposarcoma occurs with high frequency in the retroperitoneum.
- The mass appeared to be dedifferentiated liposarcoma.
- The surrounding adipose tissue with fat stranding appeared to be well-differentiated liposarcoma.

However, the diagnosis was uncertain because accumulation on FDG-PET/CT was weak in the mass.

Owing to the hypervascular mass, solitary fibrous tumour was added to the differential diagnosis. Schwannoma due to the possibility of including cystic changes, and accessory spleen due to the gradually increasing contrast enhancement effect were considered. However, there were no additional characteristic findings to suggest these were more likely than liposarcoma.

Malignant lymphoma can also sometimes occur in the retroperitoneum; however, primary malignant lymphoma is rare, and usually exhibits strong accumulation on FDG-PET/CT. Therefore, we thought that the possibility of malignant lymphoma was low.

## Treatment, outcome

Although the tumour size remained unchanged at 1-year follow-up, it was impossible to rule out malignancy; therefore, surgery was performed. The perirenal adipose tissue was peeled back from the renal capsule of the upper pole and excised en bloc with the tumour.

**Figure 4.**
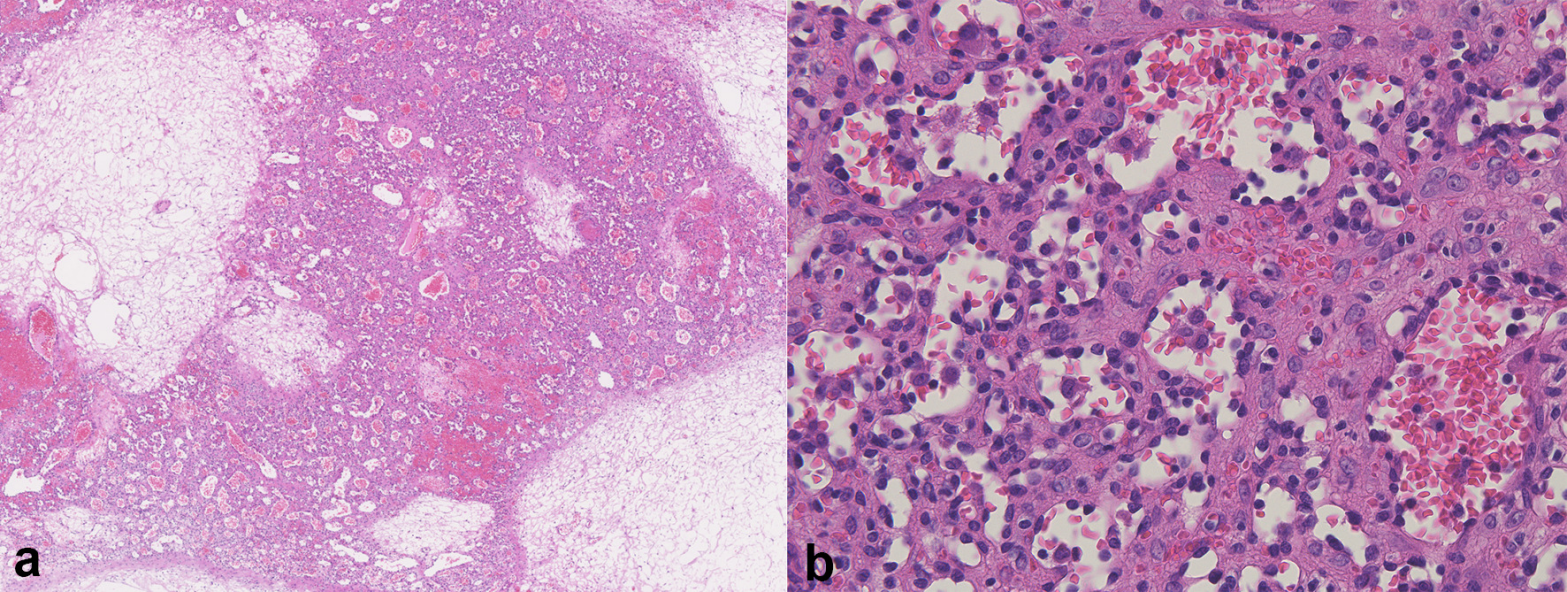
Pathological findings of anastomosing haemangioma in the perirenal space. [haematoxylin and eosin staining, original magnification (a, x25; b, x400)]. (a) The mass included a mixture of dense capillaries and mature adipose tissue. (b) The tumour was composed of anastomosing proliferation of various-sized capillary vessels that were lined with hobnail endothelial cells.

Pathologically, a brownish-coloured solid mass including a mixture of dense capillaries and mature adipose tissue was observed (Figure 4a). The pathologist did not identify any features within the tumour, such as necrosis, haemorrhage or cystic changes, which were suspected in the radiological findings. The tumour was composed of an anastomosing proliferation of various-sized capillary vessels that were lined with hobnail endothelial cells (Figure 4b). Mature adipose tissue was also observed. No mitotic activity was observed. Similar to the preoperative diagnosis, liposarcoma was considered pathologically in the differential diagnosis. On immunohistochemistry, the tumour cells were positive for p16 but negative for MDM2 nor CDK4. In addition, the amplification of *MDM2* gene was not detected in fluorescence *in situ* hybridization. Together with histopathological findings, we finally diagnosed the tumour as anastomosing haemangioma with fatty changes.

## Discussion

Anastomosing haemangioma is a rare subtype of capillary haemangioma recently defined by Montgomery et al.^1^ Anastomosing haemangioma presents with anastomosing sinusoidal capillaries in an architecture reminiscent of the splenic parenchyma. Pathologically, differentiation from angiosarcoma is problematic; however, anastomosing haemangioma does not present with mitotic activity, has little to no cellular atypia, and presents with benign pathological findings.^2^ Earlier studies have reported that it often occurs in the urogenital organs, especially the kidneys.^3–5^ John et al stated that the lesion also occurred in deep soft tissue such as the retroperitoneal adipose and paraspinal tissues.^6^

O’Neill et al reported on five cases that were well circumscribed and hyperdense on plain CT, with avid contrast enhancement and heterogeneity.^2^ “Well circumscribed” is a description consistent with well-marginated pathological findings for anastomosing haemangioma, while “hyperdense” is consistent with haemorrhage in macroscopic findings.^1,2,6–9^ Heterogeneity within the mass is believed to be due to fatty components, vascular thrombi, hyaline globules and cystic changes.^1,2^

In our case, as in other reports, anastomosing haemangioma was accompanied by fatty changes, and radiological images revealed tumour with fat stranding in the surrounding adipose tissue. Because it occurred in the retroperitoneum, it was difficult to differentiate from liposarcoma. In previous reports of anastomosing haemangioma, features were not clearly evident, although some reported cases exhibited fatty changes. In the summary of radiological findings, O’Neill et al reported fatty changes in 40% (two of five) of cases diagnosed with anastomosing haemangioma. Because of the fatty changes, past reports have described angiomyolipoma^7^ and liposarcoma^2^ in the preoperative differential diagnoses, with renal and retroperitoneal occurrence, respectively. No report has summarized a series of radiological images from a large number of cases, and there is still no report clearly describing how these fatty changes affect radiological differentiation. However, it must be recognized that clinically, retroperitoneal primary anastomosing haemangioma with fatty changes is difficult to differentiate from liposarcoma, as demonstrated in our case.

The mass in our case was also well circumscribed, with heterogeneity and findings of a gradually increasing contrast enhancement effect, similar to the report by O’Neill et al.^2^ However, a preoperative diagnosis could not be reached. Other than liposarcoma, paraganglioma, solitary fibrous tumour due to the comparatively intense staining and schwannoma due to the possibility of including cystic changes could be considered. Owing to the gradually increasing contrast enhancement effect, the differential diagnoses could also include accessory spleen, which may be convincing because of the pathological similarity. Malignant lymphoma may be the differential diagnosis in case of malignancy, or even metastasis with patients who have a history of cancer. For paraganglioma, ^123^I-metaiodobenzylguanidine scintigraphy is useful for differentiation. If FDG-PET/CT shows strong accumulation or appears to be continuous with other lesions, haemangioma can be ruled out, and a malignant lesion (malignant lymphoma or metastasis if multiple) may be more likely. However, if there is weak accumulation, findings are often non-specific and not useful for differentiation.

Percutaneous biopsy enabled O’Neill et al to diagnose the eight cases that were initially suspected to be anastomosing haemangioma; they reported no complications such as haemorrhage.^2^ If anastomosing haemangioma is strongly suspected on the basis of findings such as the site of occurrence, well-circumscribed margins, hyperdensity on plain CT, avid contrast enhancement and fatty or cystic changes, biopsy should be considered as a potential approach to avoid unnecessary surgery.

In conclusion, the anastomosing haemangioma in our case was accompanied by fatty changes with retroperitoneal occurrence; therefore, liposarcoma may be the most likely differential diagnosis. If a retroperitoneal mass is well circumscribed with avid and prolonged contrast enhancement, and includes fatty or cystic changes, anastomosing haemangioma should also be considered in the radiological differential diagnosis. In such cases, biopsy must be considered as a useful option for determining a definitive diagnosis.

## Learning points

1. Anastomosing haemangioma, which often occurs in the retroperitoneum, is well circumscribed, exhibits avid contrast enhancement, and is heterogeneous due to fatty changes; consequently, liposarcoma can be difficult to differentiate.
2. With retroperitoneal tumours accompanied by fatty changes and including a strongly enhanced area, the possibility of anastomosing haemangioma may be considered. Tumours with prolonged enhancement and minor accumulation on FDG-PET/CT may suggest anastomosing haemangioma.
3. Biopsy must be considered as a useful option for determining a definitive diagnosis.

## Consent

Written informed consent for the case to be published (including images, case history and data) was obtained from the patient(s) for publication of this case report, including accompanying images.

## Acknowledgments

We would like to thank Drs Kenji Notohara and Takashi Koyama (Department of Anatomic Pathology and Diagnostic Radiology, Kurashiki Central Hospital) for helpful discussions.
